# Progress in the total synthesis of inthomycins

**DOI:** 10.3762/bjoc.17.7

**Published:** 2021-01-07

**Authors:** Bidyut Kumar Senapati

**Affiliations:** 1 Department of Chemistry, Prabhat Kumar College, Contai, 721404, India, Tel.: +91 8145207480

**Keywords:** antibiotics, inthomycins, oxazololomycin, Stille coupling, total synthesis

## Abstract

The inthomycin family of antibiotics, isolated from *Streptomyces* strains, are interesting molecules for synthesis due to their characteristic common oxazole polyene chiral allylic β-hydroxycarbonyl fragments and significant biological activities. The full structural motif of the inthomycins is found in several more complex natural products including the oxazolomycins, 16-methyloxazolomycin, curromycins A and B, and KSM-2690. This review summarises the application of various efforts towards the synthesis of inthomycins and their analogues systematically.

## Introduction

**Figure 1:**
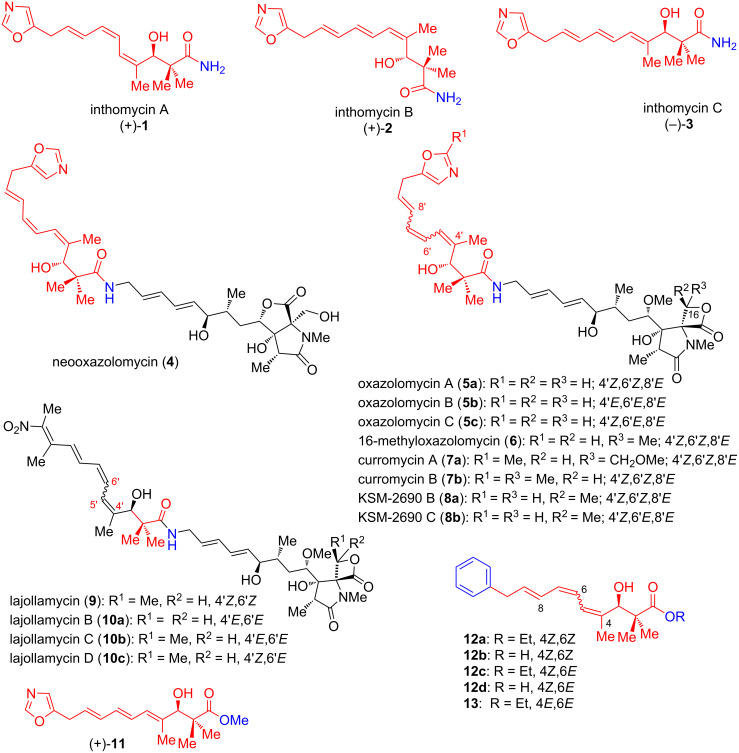
The inthomycins A–C (**1**–**3**) and structurally closely related compounds.

Inthomycins, alternatively known as phthoxazolins, are a class of compounds in which a methylene-interrupted oxazolyltriene unit is conjugated to a chiral β-hydroxycarbonyl center of an amide functionality. Inthomycin A ((+)-**1**), the ﬁrst member of the inthomycin family, was isolated by Omura’s group from the strain of *Streptomyces sp*. OM-5714 in 1990 [1]. Then, the following year, Henkel and Zeek had reported the reisolation of inthomycin A ((+)-**1**) and the first isolation of inthomycin B ((+)-**2**) from the strain of *Streptomyces sp.* Gö 2, and proved inthomycin A ((+)-**1**) to be identical with phthoxazolin A ((+)-**1**) [2]. Later, the reisolation of inthomycin B ((+)-**2**) and inthomycin C ((–)-**3**) was reported by Omura’s group in 1995 [3]. Inthomycin A ((+)-**1**) displays moderate antifungal activity against cellulose-containing *Phytophthora parasitica* and *Phytophthora capsici* [4]. Inthomycins were reported to possess many interesting biological properties, which include the specific inhibition of the cellular biosynthesis [1,4], in vitro antimicrobial activity [4–5], and anticancer activity against human prostate cancer cell lines [6–7]. A recent study suggested that the close analogue (+)-**11** of inthomycin C was found to exhibit proteasome inhibition activity [8]. The skeletal structures of inthomycins A–C (**1**–**3**) are embodied in several other naturally occurring compounds, such as neooxazolomycin (**4**), oxazolomycins A–C (**5**, **6**) [9–14], curromycins (**7**) [15], and KSM-2690 (**8**) [16] (Figure 1). Owing to their various biological activities and characteristic closely related structural motifs, they have generated immense interest among the chemists. Over the past two decades, a wide variety of synthetic strategies have been dedicated towards the synthesis of the inthomycin class of antibiotics. An earlier report on the total synthesis of oxazolomycins provides an overview of the author’s synthetic efforts toward neooxazolomycin (**4**), oxazolomycin A (**5a**), and related antibiotics [17]. The recent review of Lee has mainly focused on the application of copper(I) salt and fluoride-promoted Stille coupling reactions in the synthesis of bioactive molecules including inthomycins A–C (**1**–**3**) [18]. The present review provides a systematic summary of synthetic strategies for the synthesis of inthomycins and their analogues over the period of 1999 to present.

## Rewiew

### Synthesis

**Figure 2:**
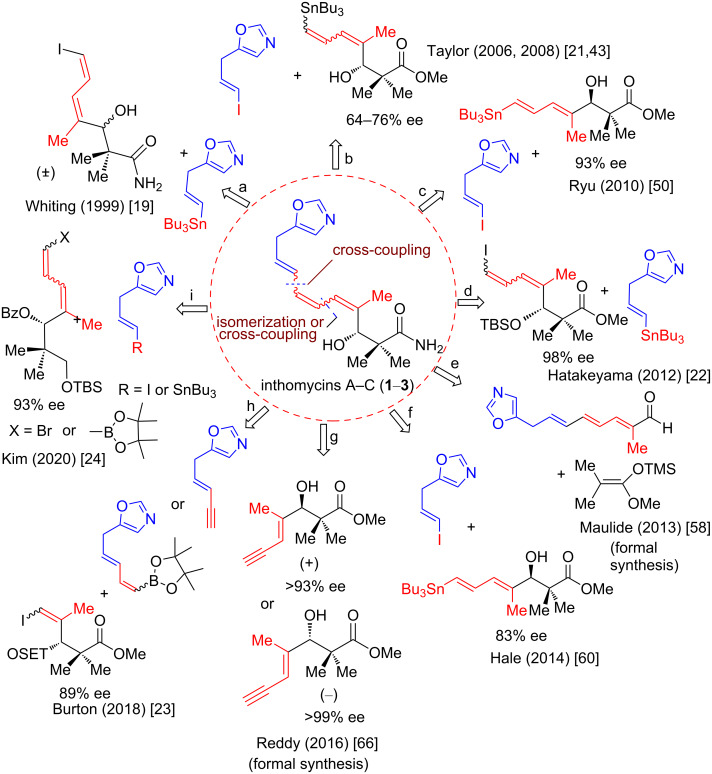
Syntheses of inthomycins A–C (**1**–**3**).

Undoubtedly, the unique skeleton of inthomycins has acted as an inspiration for the development of new synthetic methodologies. Many methods have been developed for the synthesis of inthomycins since their first isolation in 1990 [1]. Most of the reported methods have been directed towards inthomycin C (**3**) due to its thermodynamically more favored 4*E*,6*E*,8*E*-triene system. The regiochemical issues of installing the conjugated triene system, which is susceptible to *cis–trans* isomerization, have been longstanding problems in the area of inthomycins. The problems are more acute for the construction of enantioenriched β-hydroxycarbonyl units as evident from the recent reports [19–21]. Since the pioneering works of Henaff and Whiting [19–20], several racemic and asymmetric total syntheses of inthomycins A–C (**1**–**3**) have been carried out in many research groups (Figure 2). However, only four synthetic strategies that lead to the total synthesis of all three members of inthomycins A–C (**1**–**3**) are available (Figure 2, route b, d, h, and i) [21–24].

**Scheme 1:**
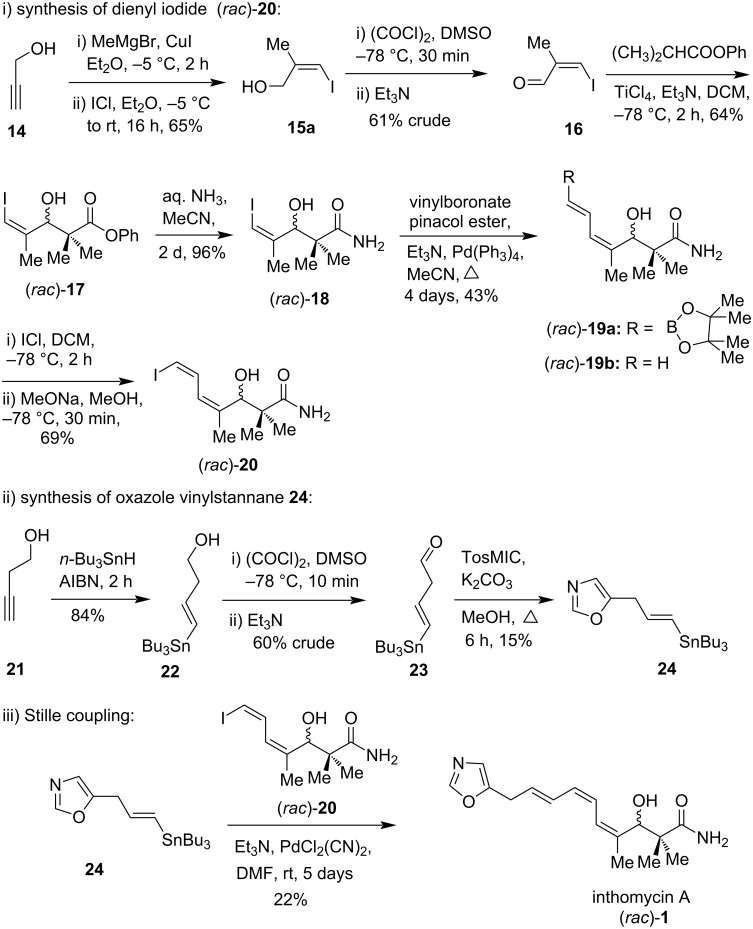
The first total synthesis of racemic inthomycin A (*rac*)-**1** by Whiting.

The first racemic synthesis of inthomycin A ((*rac*)-**1**), alternatively known as phthoxazolin A, was reported in 1999 [19]. The key steps include i) the synthesis of intermediate dienyl iodide (*rac*)-**20**, ii) the synthesis of intermediate oxazole vinylstannane **24**, and iii) the Stille coupling between oxazole vinylstannane **24** and dienyl iodide (*rac*)-**20** as the final step. The synthesis began with alcohol (*Z*)-**15a**, which was readily prepared in 65% yield from propargyl alcohol (**14**) by using a copper(I)-catalyzed methyl Grignard addition followed by in situ iodinolysis. The Swern oxidation of (*Z*)-**15a** followed by immediate aldol condensation afforded racemic phenol ester (*rac*)-**17** via aldehyde **16**. However, several attempts to form enantioenriched aldol fragment **18** using both a chiral auxiliary [25–28] and catalytic asymmetric [29–30] procedures proceeded without success. Therefore, the synthesis of inthomycin A was advanced in the racemic form. Treatment of compound (*rac*)-**17** with aqueous ammonia gave the corresponding amide (*rac*)-**18** in 96% yield. Heck coupling between (*rac*)-**18** and vinylboronate pinacol ester [31] using Pd(PPh_3_)_4_/Et_3_N conditions provided stereoselective access to dienylboronate (*rac*)-**19a** in 43% yield. A competitive Suzuki coupling was also observed with compound (*rac*)-**19b** being isolated in 35% yield [32–35]. Dienylboronate (*rac*)-**19a** was then transformed into dienyl iodide (*rac*)-**20** by iodine monochloride addition and methoxide-mediated elimination (Scheme 1) [35]. The oxazole vinylstannane **24** was prepared from commercially available butyne **21**. The tri-*n*-butyltin hydride addition to **21**, followed by Swern oxidation and direct oxazole formation with tosylmethyl isocyanide (TosMIC) gave the fragment **24**, which was used immediately for the next step due to its high instability. Finally, the Stille cross-coupling reaction between vinylstannane **24** and dienyl iodide (*rac*)-**20** using PdCl_2_(CN)_2_ and triethylamine in DMF produced racemic inthomycin A (or phthoxazolin A) (*rac*)-**1** in 22% yield (Scheme 1).

Although the overall yield of this route was very low, this work certainly established the basis for the future enantioselective syntheses of inthomycins and related natural products.

**Scheme 2:**
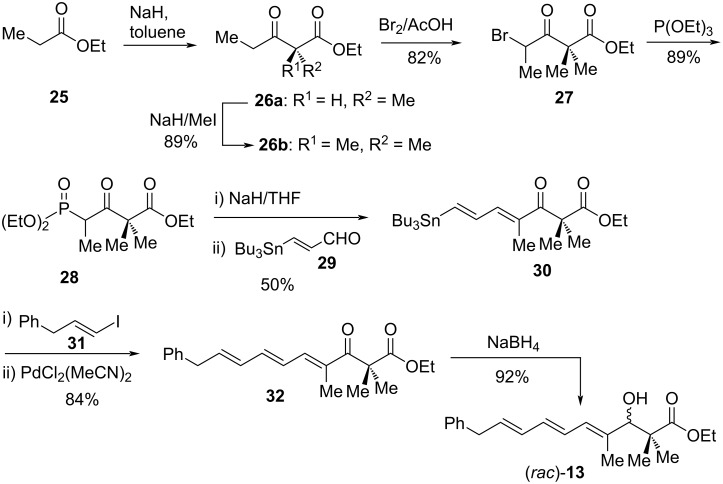
Moloney’s synthesis of the phenyl analogue of inthomycin C ((*rac*)-**3**).

In 2002, Moloney et al. described an eﬃcient synthetic route using the Stille coupling reaction as the key step to accomplish the synthesis of phenyl analogues of inthomycins [36]. These triene moieties are a sub-unit of the oxazolomycin class of antibiotics. To prepare the phenyl analogue of racemic inthomycin C (*rac*-**3**), at first, the phosphonate **28** was prepared using a Claisen condensation of ethyl propionate (**25**) followed by methylation of **26a**, treatment with bromine in acetic acid, and then triethyl phosphite. Next, compound **28** was treated with sodium hydride followed by aldehyde **29** [37] to give (*E,E*)-dienyl stannane **30** in 50% yield. The key Stille coupling between **30** and vinyl iodide **31**, prepared by Takai reaction [38] of phenylacetaldehyde, in presence of PdCl_2_(MeCN)_2_ produced (*E,E,E*)-triene **32** in 84% yield. Finally, NaBH_4_ reduction of **32** gave alcohol (*rac*)-**13**, in which the triene moiety is analogous to inthomycin C ((*rac*)-**3**) and oxazolomycin B (**5b**) (Scheme 2).

**Scheme 3:**
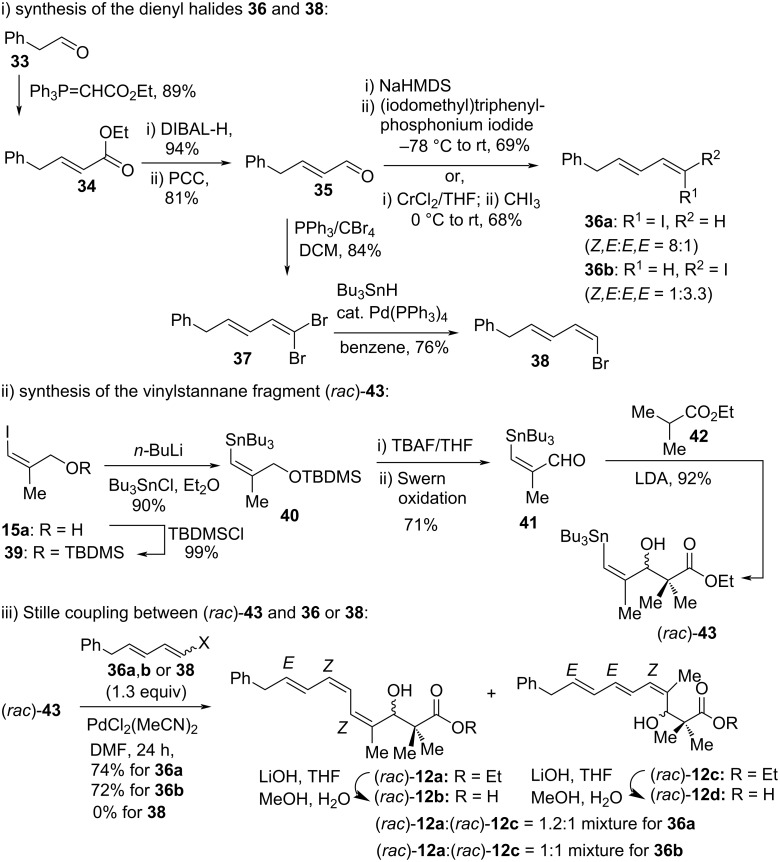
Moloney’s synthesis of phenyl analogues of inthomycins A (*rac*-**1**) and B (*rac*-**2**).

After the successful application of the Stille reaction to construct the (*E,E,E*)-triene system (*rac*)-**13** in a stereoselective manner, attention was then focused on the development of an analogous strategy towards the (*Z,Z,E*)- and (*Z,E,E*)-triene systems present in oxazolomycin A (**5a**) and oxazolomycin C (**5c**), respectively. The key steps were i) synthesis of dienyl halides, ii) synthesis of the required vinylstannane and iii) Stille coupling between them (Scheme 3) [39]. The required divinyl halides **36** were prepared, starting from phenylacetaldehyde (**33**), by using the Takai [38] or Wittig procedures [40] as shown in Scheme 3 (68% of a 3.3:1 mixture of (*E*,*E*)/(*Z*,*E*)-**36b** and 69% yield of a 8:1 mixture of (*Z*,*E*)/(*E*,*E*)-**36a**, respectively). Aldehyde **35** was then converted into dibromide **37** using PPh_3_/CBr_4_ followed by stereoselective palladium-catalyzed monoreduction according to the literature available protocol [41] to give vinyl bromide **38** in 76% yield (*Z*/*E* as 99:1 mixture). Iodide **15a** [42] was prepared stereoselectively from propargyl alcohol following the literature procedure, and the free hydroxy group was then protected as its TBDMS ether to produce **39** in 99% yield.

The metal–halogen exchange of **39** followed by the Bu_3_SnCl quench in Et_2_O gave the desired stannane **40** in excellent yield (90%). Deprotection of the TBDMS ether of stannane **40** with tetra-*n*-butylammonium fluoride (TBAF) in THF and then subsequent Swern oxidation of the crude alcohol gave aldehyde **41** in 71% yield. The aldehyde **41** was treated with ethyl isobutyrate (**42**) in the presence of LDA to afford the aldol adduct (*Z*)-(*rac*)-**43** in the racemic form (92% yield). The stannane (*Z*)-(*rac*)-**43** was then subjected to Stille coupling with iodide (*E*,*E*)-**36b** (3:1 mixture of stereoisomers, 3 equiv) using Pd(CH_3_CN)_2_Cl_2_/DMF conditions to produce a 3:1 mixture of (*Z,Z,E*):(*Z,E,E*) triene products (*rac*)-**12a** and (*rac*)-**12c** in 72% yield after 10 h reaction time. After optimization, it was found that the above product ratio changed to 1:1 when the reaction was allowed to run for 24 h and only 1.3 equiv of (*E,E*)-**36b** was employed in the Stille coupling. In a similar manner, stannane (*Z*)-**43** coupled with (*Z,E*)-iodide **36a** (8:1 ratio of isomers, 1.3 equiv) to produce a 1.2:1 mixture of (*Z,Z,E*):(*Z,E,E*) triene products (*rac*)-**12a** and (*rac*)-**12c** in 74% yield. The stereoisomers (*rac*)-**12a** and (*rac*)-**12c** were found to be inseparable by chromatography. The isomerically pure bromide (*Z*)-**38** was found to be inert to coupling with vinylstannane (*rac*)-**43** under standard Stille conditions. Hydrolysis of a mixture (3:1) of (*rac*)-**12a** and (*rac*)-**12c** produced a mixture (3:1) of the corresponding acids (*rac*)-**12b** and (*rac*)-**12d** in 70% yield (Scheme 3). These trienes are analogues of oxazolomycins A (**5a**) and C (**5c**) and inthomycins A (*rac*-**1**) and B (*rac*-**2**), respectively.

**Scheme 4:**
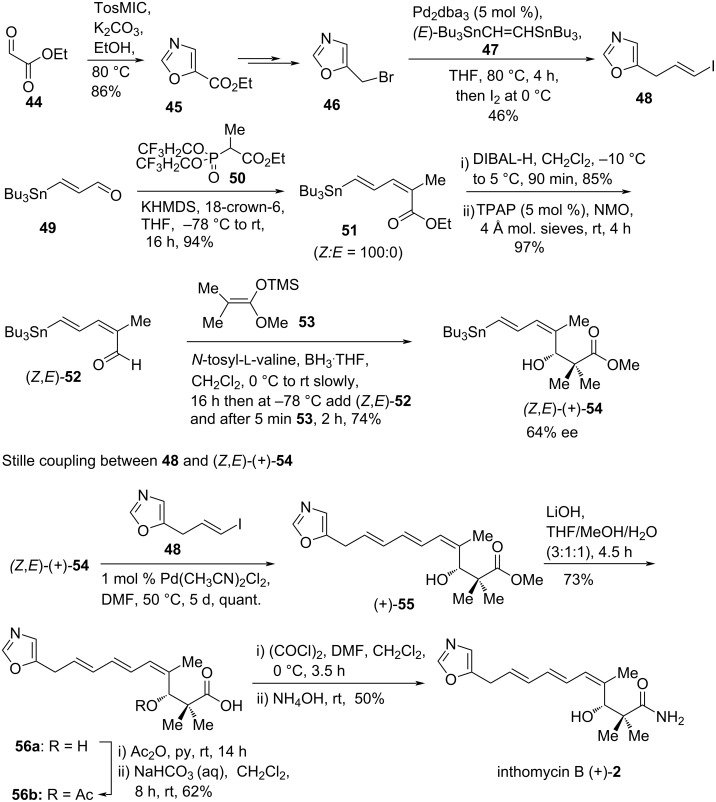
The first total synthesis of inthomycin B (+)-**2** by R. J. K. Taylor.

In 2006, R. J. K. Taylor and co-workers reported the first total synthesis of inthomycin B ((+)-**2**) using a Stille coupling of a stannyl-diene with an oxazole vinyl iodide unit followed by a Kiyooka ketene acetal/amino acid-derived oxazaborolidinone procedure as its cornerstones (Scheme 4) [43]. In the beginning, oxazole **45** was prepared in good yield (86%) by treating ethyl glyoxylate with tosyl methyl isocyanate (TosMIC) in the presence of K_2_CO_3_ at 80 °C [44]. The reduction of the ethyl ester of **45** followed by NBS treatment gave unstable bromide **46** [45], which was immediately coupled to (*E*)-1,2-bis(tri-*n*-butylstannyl)ethene (**47**) using catalytic Pd_2_dba_3_ in refluxing THF to produce iodide **48** in 46% yield. The coupling partner (*Z,E*)-(+)-**54** was prepared enatioselectively from the known (*E*)-3-(tributylstannyl)propenal (**49**) [46] using a four-step sequence. Treatment of **49** with the Still–Gennari bis-trifluoroethoxy phosphonate reagent **50** proceeded stereoselectively to give ester **51** in excellent yield (94%). Subsequent DIBAL-H reduction of ester **51** followed by tetrapropylammonium perruthenate (TPAP) oxidation afforded aldehyde (*Z,E*)-**52** as a single isomer in 83% yield over two steps. The asymmetric aldol reaction aldehyde (*Z,E*)-**52** with silyl ketene acetal **53** in the presence of oxazaborolidinone derived from *N*-tosyl-ʟ-valine and BH_3_·THF generated the desired alcohol (*Z,E*)-(+)-**54** in 74% yield and 64% ee. Next, a wide range of catalysts/conditions were screened for the crucial Stille coupling between iodide **48** and (*Z,E*)-(+)-**54** to overcome the problems of isomerization of the (*Z,E,E*)-triene unit of the desired products. Finally, PdCl_2_(CH_3_CN)_2_ (1 mol %) in DMF was found to smoothly deliver the required triene (+)-**55** in quantitative yield. After many unsuccessful attempts of direct conversion of methyl ester (+)-**55** into the corresponding primary amide, acetylation of acid **56a** followed by acid chloride formation of acetate **56b** and in situ ammonium hydroxide treatment was found to be fruitful to produce inthomycin B (+)-**2** in reasonable yield (Scheme 4). This synthetic route introduced the chiral entry to any member of the inthomycin family for the first time.

**Scheme 5:**
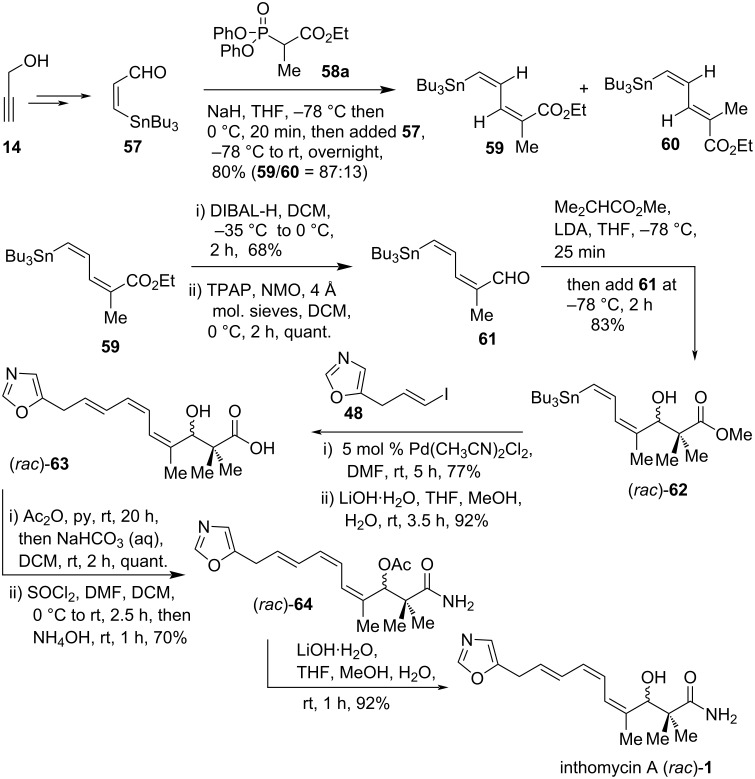
R. J. K. Taylor’s total synthesis of racemic inthomycin A (*rac*)-**1**.

**Scheme 6:**
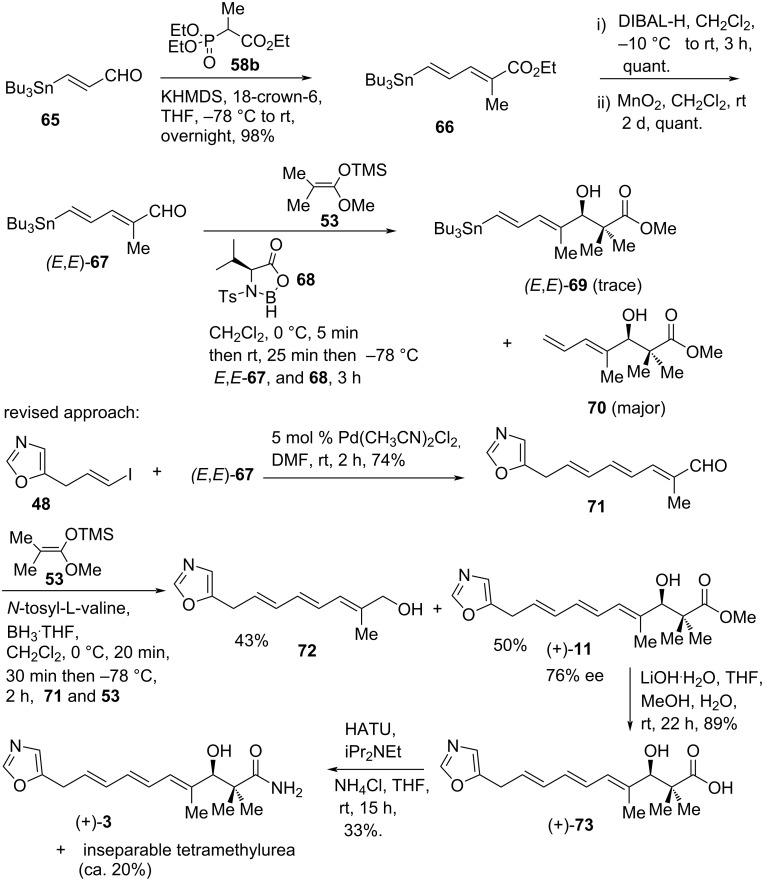
The first total synthesis of inthomycin C ((+)-**3**) by R. J. K. Taylor.

In 2008, R. J. K. Taylor and co-workers described a unified synthetic route to the inthomycin family [21]. The developed procedure has been utilized successfully to prepare racemic inthomycin A ((*rac*)-**1**) and inthomycin C ((+)-**3**) (Scheme 5 and Scheme 6). The previously reported procedure [20,45] was modified to improve the yield of inthomycin A ((*rac*)-**1**) by replacing the Stille coupling partners. The required (*Z*)-3-(tributylstannyl)propenal (**57**) was easily accomplished by LiAlH_4_ reduction of propargyl alcohol (**14**) and then transmetallation with Bu_3_SnCl followed by oxidation of the resulting alcohol using the Ley-Griffith TPAP procedure [47–48]. Treatment of aldehyde **57** with the Ando phenoxy phosphonate **58a** [49] gave the desired (*Z,Z*)-diene **59** as the major product (**59**/**60** = 87:13). DIBAL-H reduction of **59** allowed the isolation of (*Z,Z*)-isomeric alcohol followed by subsequent oxidation produced aldehyde (*Z,Z*)-**61**. After extensive unsuccessful efforts to produce enantiopure aldol fragment (*Z,Z*)-**62** using the *N*-tosyl-ʟ-valine-derived oxazaborolidinone, the racemic synthesis of (*Z,Z*)-(*rac*)-**62** was achieved by utilizing the lithium enolate of methyl isobutyrate. Stille coupling of (*Z,Z*)-(*rac*)-**62** with vinyl iodide **48** in the presence of Pd(CH_3_CN)_2_Cl_2_ in DMF proceeded smoothly to give the corresponding ester (triene isomerization was less than 20% during coupling), which was then converted into acid derivative (*rac*)-**63** by saponification. Finally, the acid derivative (*rac*)-**63** was subjected to undergo acetylation, acid chloride formation, and quenching with ammonium hydroxide to produce amide derivative (*rac*)-**64** in 70% yield. Finally, saponification of acetate (*rac*)-**64** using lithium hydroxide gave racemic inthomycin A ((*rac*)-**1**) in 14% overall yield (Scheme 5).

The total synthesis of inthomycin C ((+)-**3**) was achieved by using a Stille coupling between (*E,E,*)-**67** and vinyl iodide **48** followed by directed asymmetric aldol reaction under Mukaiyama–Kiyooka aldol reaction conditions (Scheme 6). Initially, (*E*)-3-(tributylstannyl)propenal (**65**) was converted into (*E,E*)-diene **66** (*E*/*Z* = 19:1, separable) using the standard (*E*)-selective Horner–Wadsworth–Emmons (HWE) reaction. DIBAL-H reduction of ester **66** followed by MnO_2_ oxidation produced aldehyde (*E,E*)-**67** stereoselectively. Unfortunately, attempted enantioselective aldol reactions of (*E,E*)-**67** with silylketene acetal **53** using *N*-tosyl-ʟ-valine-derived oxazaborolidinone **68** gave a negligible amount of the required dienylstannane (*E,E*)-**69** with a significant amount of destannylated product **70**. The destannylation process could not be prevented even after a range of reaction conditions were tested. Thus, this approach was revised and the key Stille coupling was carried out before the introduction of the asymmetric aldol fragment (Scheme 6). Therefore, the key Stille coupling of aldehyde (*E,E*)-**67** with vinyl iodide **48** in the presence of PdCl_2_(CH_3_CN)_2_ gave (*E,E,E*)-trienal **71** in 74% yield. The trienal **71** underwent asymmetric aldol reaction in the presence of oxazaborolidinone derivative **68** and silyl ketene acetal **53** to produce the required α-hydroxy ester (+)-**11** in 50% yield and 76% ee ((*R*)-stereochemistry of the major enantiomer). A competitive reduction of **71** was also observed to produce alcohol **72** in 43% yield. Finally, hydrolysis of the ester (+)-**11** followed by hexafluorophosphate azabenzotriazole tetramethyl uranium (HATU)-mediated coupling with ammonia gave inthomycin C ((+)-**3**) in 33% yield, containing inseparable tetramethylurea as byproduct (ca. 20%).

**Scheme 7:**
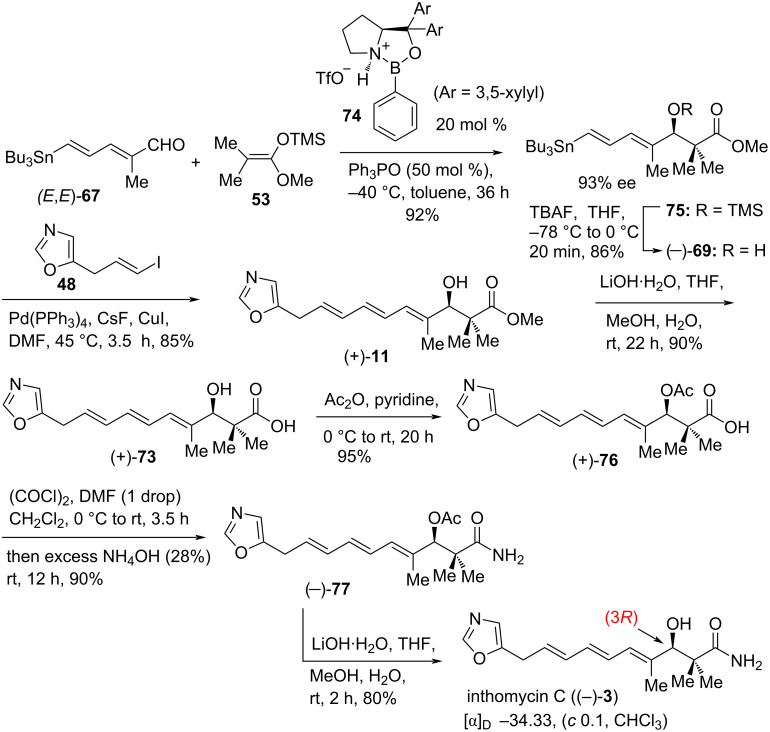
The first total synthesis of naturally occurring inthomycin C ((–)-**3**) by Ryu et al.

In 2010, Senapati, Ryu et al. [50] described the first total synthesis of naturally occurring inthomycin C ((–)-**3**) in excellent yield and enantiopurity by employing a cationic oxazaborolidinium-catalyzed asymmetric Mukaiyama aldol reaction and Stille coupling as the key steps (Scheme 7). Treatment of compound **75** with tetra-*n*-butylammonium fluoride (TBAF) in THF at −78 °C and the resulting solution was carefully allowed to warm to 0 °C to give the desired alcohol (*E*,*E*)-(−)-**69** in 86% yield and 93% ee with only trace amounts of the corresponding destannylated product, a major disadvantage of R. J. K. Taylor’s synthesis of (+)-inthomycin C, ((+)-**3**, see Scheme 6, compound **70**) [21]. Next, the key Stille coupling reaction of dienylstannane (*E*,*E*)-(–)-**69** with oxazole vinyl iodide **48** using Pd(PPh_3_)_4_/CsF/CuI conditions [51–52] gave ester (+)-**11** in 85% yield. The ester (+)-**11** was then hydrolyzed with lithium hydroxide to give the corresponding acid (+)-**73** in 90% yield. The transformation of acid (+)-**73** to acetate (+)-**76** using acetic anhydride in pyridine followed by acid activation with oxalyl chloride and then in situ treatment with 28% ammonium hydroxide afforded amide (−)-**77** in 90% yield. Finally, deacetylation of (−)-**77** using lithium hydroxide produced (−)-inthomycin C ((−)-**3**) in 80% yield with high enantiopurity (Scheme 7).

**Scheme 8:**
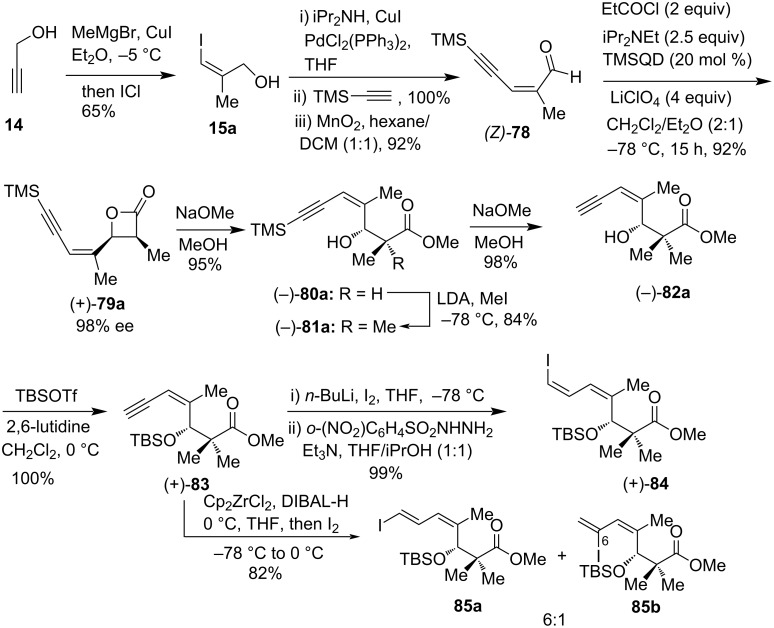
Preparation of *E*,*E*-iododiene (+)-**84** and *Z*,*E*- iododiene **85a**.

**Scheme 9:**
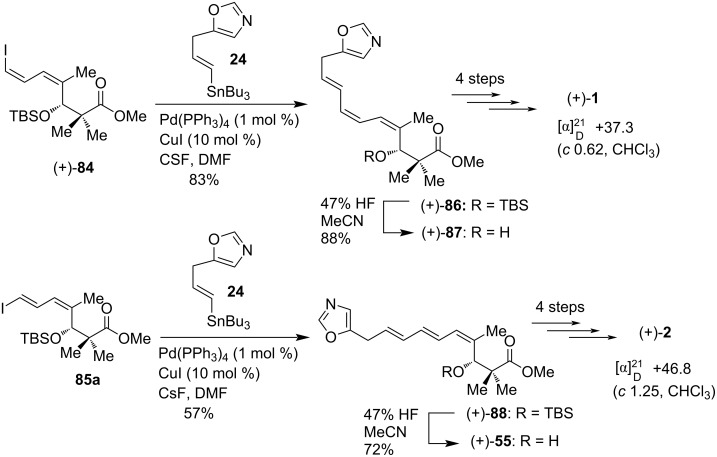
Hatakeyama’s total synthesis of inthomycin A (+)-**1** and inthomycin B (+)-**2**.

**Scheme 10:**
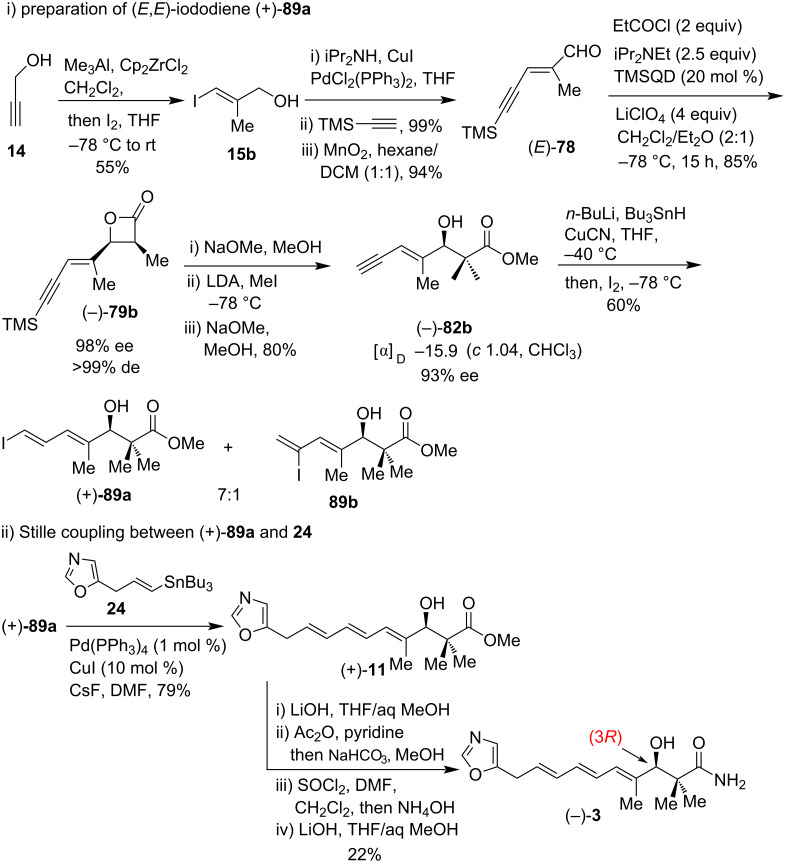
Hatakeyama’s total synthesis of inthomycin C ((–)-**3**).

In 2012, Hatakeyama and co-workers reported a unified strategy for the asymmetric total syntheses of inthomycin A ((+)-**1**), inthomycin B ((+)-**2**), and inthomycin C ((−)-**3**), starting with an organocatalytic asymmetric [2 + 2] cycloaddition reaction of an aldehyde and a ketene followed by their isomerization-free Stille coupling with (*E*)-5-(3-(tributylstannyl)allyl)oxazole (Schemes 8–10) [22]. In this synthesis, the enantiopure β-lactone (+)-**79a** was synthezised from (*Z*)-aldehyde **78** and propionyl chloride according to Nelson’s method [53–55] using quinidine TMS ether (TMSQD), LiClO_4_, and Hünig’s base. The aldehyde (*Z*)-**78** was obtained from propargyl alcohol **14** via (*Z*)-iodo alcohol **15a**. The β-lactone (+)-**79a** was converted to enantioenriched vinyl iodides (+)-**84** and **85a** separately. Thus, methanolysis of (+)-**79a** provided (–)-**80a** which was methylated using methyl iodide and lithium diisopropylamide (LDA) to produce (–)-**81a** in 84% yield. Desilylation of (–)-**81a** followed by *tert*-butyldimethylsilyl (TBS) protection of (–)-**82a** gave ester (+)-**83**. Compound (+)-**83** was converted to (*Z,Z*)-(+)-**84** by using iodination and a diimide reduction as reported previously [56]. Similarly, the treatment of (+)-**83** with in situ-generated Schwartz’s reagent from zirconocene dichloride and DIBAL-H followed by iodine to produce an inseparable 6:1 mixture of (*Z,E*)-iododiene **85a** and its 6-iodo-isomer **85b** in 82% yield (Scheme 8).

The vinyl iodide (*Z,Z*)-(+)-**84** was coupled to stannane **24**, using the Mee–Lee–Baldwin (MLB) protocol [51–52], to aﬀord (+)-**86**. Finally, removal of TBS protection followed by functional group modifications, compound (+)-**86** was transformed into inthomycin A ((+)-**1**). Similarly, the coupling between vinyl iodide **85a** and stannane **24** produced triene (+)-**88**. Subsequently, compound (+)-**55** was transformed into inthomycin B ((+)-2, Scheme 9).

For the synthesis of inthomycin C ((−)-**3**), Hatakeyama et al. prepared (*E*)-aldehyde **78** starting from propargyl alcohol **14** via (*E*)-iodo alcohol **15b**. Treatment of (*E*)-**78** with propionyl chloride, LiClO_4_, and diisopropylethylamine in the presence of quinidine TMS ether (TMSQD) according to Nelson’s procedures [53–55] provided the enantioenriched β-lactone (−)-**79b** in 85% yield. The desired terminal acetylene (−)-**82b** was synthesized from β-lactone (−)-**79b** following a three-step sequence as shown in Scheme 10. Subsequent stannylation followed by iodination converted compound (−)-**82b** to an inseparable 7:1 mixture of (*E,E*)-iododiene (+)-**89a**, and its 6-iodo isomer **89b** in 60% yield. Compound (+)-**89a** was then subjected to Stille coupling with oxazole vinylstannane **24** using Pd(PPh_3_)_4_, CuI, and CsF in DMF at room temperature to give (*E,E,E*)-(+)-**11** in 79% yield. This compound was then transformed successfully to (inthomycin C ((−)-**3**) in a four-step sequence (Scheme 10).

The diﬀerence in Hatakeyama’s approach to inthomycin C ((–)-**3**) is the polarity reversal of the Stille coupling components relative to that from R. J. K. Taylor’s and Ryu’s study (see Scheme 6 and Scheme 7). Hatakeyama et al. reported the specific rotation of their synthetic inthomycin C ((–)-**3**) as [α]_D_ −41.5 (*c* 0.10, CHCl_3_) [22] whereas R. J. K. Taylor and Ryu reported the values as [α]_D_ +25.9 (*c* 0.27, CHCl_3_, containing ca. 20% inseparable tetramethylurea) [21] and [α]_D_ −34.33 (*c* 0.10, CHCl_3_) [50], respectively. The specific rotation values provided by Hatakeyama and Ryu’s groups were consistent with respect to their synthetic (3*R*)-inthomycin C ((–)-**3**). Although, this [α]_D_ measurement was found to be in the opposite sign to the value reported by the group of R. J. K. Taylor. Therefore, the absolute configuration assignment of (3*R*)-inthomycin C ((–)-**3**) as described by the Ryu and Hatakeyama groups contradicted with that of the R. J. K. Taylor group. Later, the groups of Hale and Hatakeyama tried hard to eliminate all the contradictions regarding the specific rotation values of inthomycin C and securely assigned the (3*R*)‑configuration for inthomycin C ((−)-**3**) [57].

**Scheme 11:**
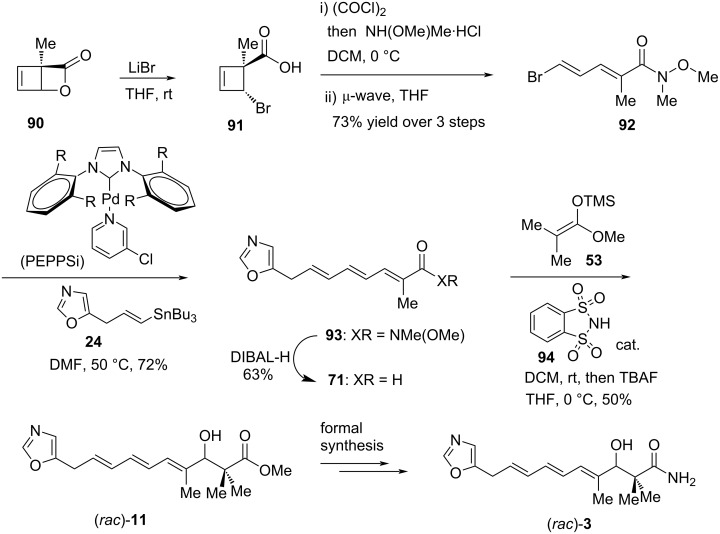
Maulide’s formal synthesis of racemic inthomycin C ((*rac*)-**3**).

In the synthetic studies towards racemic inthomycin C ((*rac*)-**3**), Maulide and co-workers investigated the stereoselective synthesis of halocyclobutenes and their ring-opening reactions (Scheme 11) [58]. The synthesis commenced with the smooth ring opening of the methyl-substituted lactone **90** using lithium bromide, which gave a single *trans*-cyclobutenyl bromide **91**. Then, the bromocyclobutene **91** was submitted to further amide coupling and 4π electrocyclic ring opening to produce 2-methyl-5-bromodienoic amide **92** stereoselectively in 73% yield over three steps. Stille cross-coupling of **92** with vinylstannane **24** followed by DIBAL-H reduction produced aldehyde **71**, which then underwent an organocatalytic Mukaiyama aldol reaction with silylketene acetal **53** to produce racemic (*E,E,E*)-triene (*rac*)-**11** in 50% yield [59]. Since triene (*rac*)-**11** has been previously transformed into inthomycin C, this study demonstrated the formal synthesis of racemic inthomycin C ((*rac*)-**3**) [21–22,43,50].

**Scheme 12:**
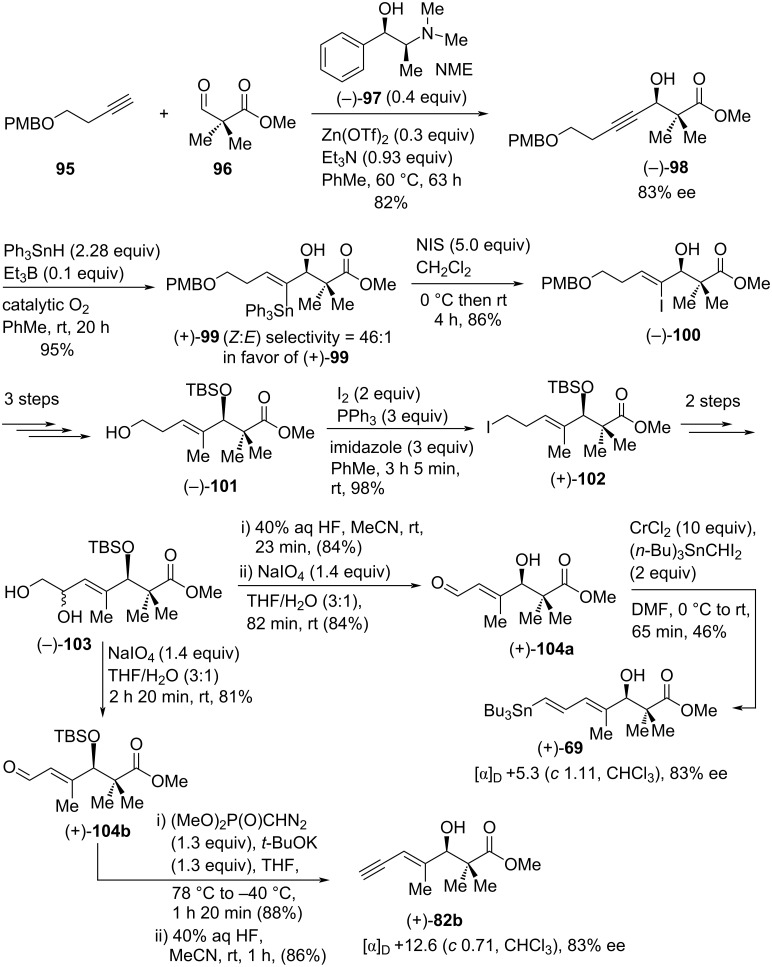
Hale’s synthesis of dienylstannane (+)-**69** and enyne (+)-**82b** intermediates.

**Scheme 13:**
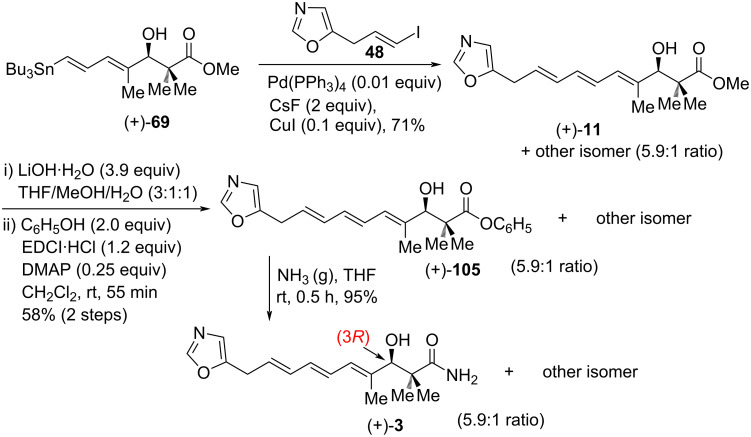
Hale’s total synthesis of inthomycin C ((+)-**3**).

An interesting application of the O-directed free radical hydrostannation reaction was demonstrated by Hale et al. in the total synthesis of inthomycin C ((+)-**3**) and it was also claimed that the previous (3*S*)-stereochemical revision of inthomycin C ((+)-**3**) by the groups of Ryu [50] and Hatakeyama [22] was found to be invalid based on their modified Mosher ester preparation and optical rotation evidence [60]. The synthetic route was designed in such a way that intercept both Ryu’s intermediate (+)-**69** [50] and Hatakeyama’s intermediate (+)-**82b** [22] (Scheme 12 and Scheme 13). In this approach, the asymmetric alkylation of **96** with alkyne **95** under Carreira’s conditions [61–63] afforded (−)-**98** in 82% yield (83% ee). Subsequent free radical hydrostannation on (−)-**98** produced (+)-**99** as the major product of a 46:1 mixture of (*Z*/*E*)-α-stannylated geometric isomers. The puriﬁed vinylstannane (+)-**99** underwent iodination stereoselectively with excess *N*-iodosuccinimide to give (−)-**100**, which was then transformed into (−)-**101** using a three-step sequence. Upon iodination of (−)-**101** produced iodide (+)-**102** in excellent yield. Deiodination of (+)-**102** followed by regioselective dihydroxylation with Sharpless’ AD mix-β reagent [64–65] provided diol (−)-**103** as a mixture of stereoisomers. Significantly, the diol (−)-**103** was transformed into both enantiopure fragments (+)-**69** and (+)-**82b** successfully (Scheme 12).

The observed specific rotation value for (+)-**69** was found to be +5.3° (*c* 1.11, CHCl_3_) [60], which contradict the previous results ([α]_D_ −17.5° (*c* 0.12, CHCl_3_) [50]. At this stage, Hale and co-workers had concluded that (+)-**69** must have (3*R*)-stereochemistry and they had completed a formal total synthesis of the Zeeck−Taylor [2,21] stereostructure for inthomycin C ((+)-**3**). To remove any doubt, Stille cross-coupling of **48** with (+)-**69** was performed under Ryu’s conditions [50] to give the desired product (+)-**11** with a 5.9:1 mixture of inseparable stereoisomeric triene components. Compound (+)-**11** was then smoothly converted into a 5.9:1 mixture of inthomycin C ((+)-**3**) and another isomer via a three-step sequence (Scheme 13).

Unfortunately, further purification of (+)-**3** could not be achieved at the end and the [α]_D_ value obtained for the 5.9:1 mixture of inthomycin C ((+)-**3**) was −8.4 (*c* 1.0, CHCl_3_). The observed [α]_D_ value was lower in magnitude compared to that of Ryu’s [50] and Hatakeyama’s synthesis [22] and opposite in sign with R. J. K. Taylor’s synthesis [21]. On the other hand, the specific rotation value of the newly synthesized fragment (+)-**82b** (see Scheme 12) had a similar magnitude but opposite in sign to that reported by Hatakeyama previously [22]. Thus, the absolute stereochemical assignment of enyne (+)-**82b** was revised as (3*R*), which contradicted Hatakeyama’s postulated (3*R*) configuration of enyne (−)-**82b** (Scheme 10) [22]. Hence, this new total synthesis has claimed to reinstate the originally formulated Zeeck–Taylor (3*R*)-stereostructure [2,21] for inthomycin C ((+)-**3**), R. J. K. Taylor’s total synthesis (Scheme 6) and disputes Ryu and Hatakeyama’s (3*S*)-stereochemical revision of inthomycin C (+)-**3** (See Scheme 7 and Scheme 10).

**Scheme 14:**
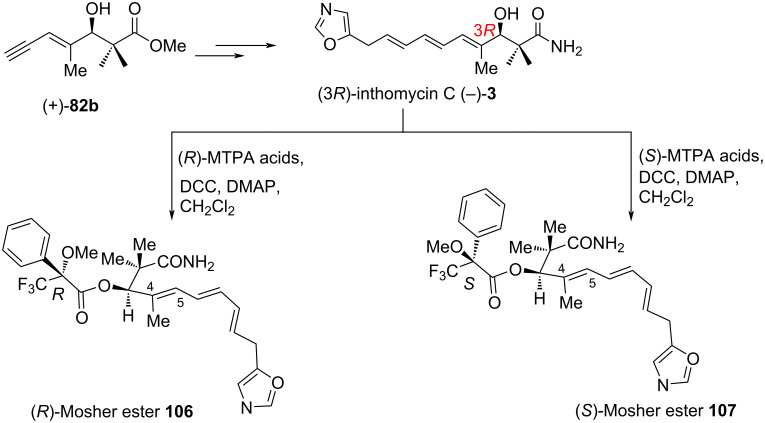
Hale and Hatakeyama’s resynthesis of (3*R*)-inthomycin C (−)-**3** Mosher esters.

Soon after, a subsequent collaboration between the Hale and Hatakeyama groups demonstrated that inthomycin C ((–)-**3**) has (3*R*)- and not (3*S*)-stereochemistry [57]. Careful reappraisal of the previously published work [21–22,50,60] now strongly recommends that the R. J. K. Taylor, Ryu, Hatakeyama, and Hale teams all have synthesized (3*R*)-inthomycin C ((−)-**3**), despite their discrepant [α]_D_ values. All the disputes and anomalies regarding the specific rotation values of the common intermediate **82b** and the synthetic (3*R*)-inthomycin C described by Hale and others were finally resolved in this work. Subsequently, the Hatakeyama team converted their authentic (+)-**82** into the (*R*)- and (*S*)-MTPA Mosher esters which provided well-matched NMR data to Hale’s previous synthesis [60]. Upon further purification using flash chromatography, the Hatakeyama team re-examined their [α]_D_ data for the authentic (3*R*)-enynol **82b** which had been stored in their laboratory and it was found that (3*R*)-**82b** had a [α]_D_ of +12.2 (*c* 0.95, CHCl_3_). This specific rotation was well consistent with the +12.6 (*c* 0.71, CHCl_3_) value as reported by the Hale group previously for the same enynol **82b** (Scheme 12). Then, the Hatakeyama and Hale groups worked together to resolve the disagreement regarding [α]_D_ values of (3*R*)-**82b** that had been published by both groups. As a part of their collaborative work, the Hale group collected the purified (3*R*)-enynol **82b** from the Hatakeyama team and recorded its specific rotation value as +14.4 (*c* 0.58, CHCl_3_). Following this correct [α]_D_ measurement, both groups concurred that they had prepared the same (3*R*)-enynol **82b** and indeed completed the total synthesis of (3*R*)-inthomycin C ((−)-**3**) (Scheme 10 and Scheme 12). Also, the Hatakeyama group freshly resynthesized inthomycin C from their remaining sample of the (+)-(3*R*)-**82b** and subsequent [α]_D_ measurement provided a value of −7.9 (*c* 0.33, CHCl_3_) (Scheme 14). Although this new value was much lower in magnitude compared to previous reports (−41.5 (*c* 0.1, CHCl_3_)) [22] by the same group, it was much closer to Hale’s report [60] for a 5.9:1 mixture of inthomycin C (−8.4 (*c* 1.0, CHCl_3_)) (see Scheme 13). Meanwhile, the Hale group transformed the resynthesized inthomycin C ((−)-**3**) into the corresponding (*R*)-and (*S*)-MTPA ester derivatives **106** and **107**, respectively (Scheme 14). Based on the corrected specific rotation value of inthomycin C ((−)-**3**) and re-investigation of their (*R*)- and (*S*)-MTPA esters, Hale and Hatakeyama jointly concluded that Ryu and others had indeed synthesized (3*R*)-inthomycin C ((−)-**3**).

**Scheme 15:**
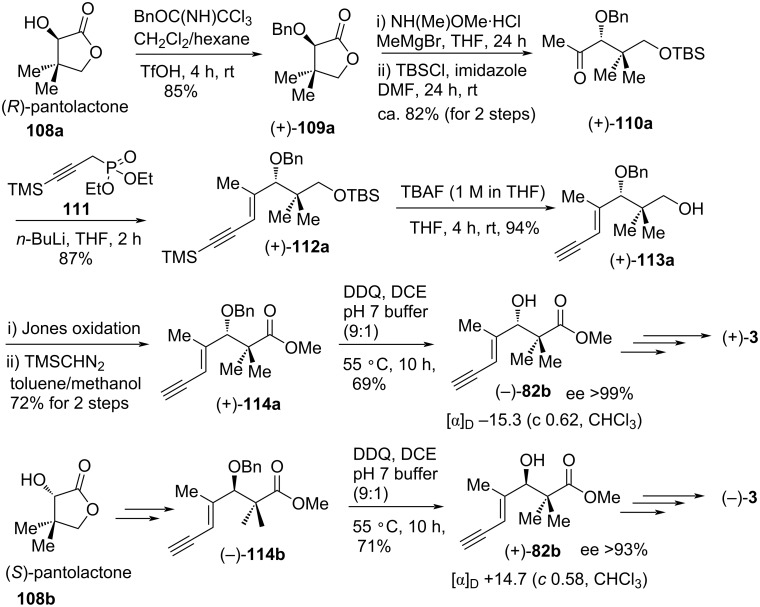
Reddy’s formal syntheses of inthomycin C (+)-**3** and inthomycin C ((−)-**3**).

Two years later, Reddy and co-workers accomplished enantiospecific formal syntheses of both inthomycins C ((+)-**3**) and inthomycin C ((−)-**3**) using Hatakeyama’s enynol intermediate **82b** (Scheme 15) [66]. The synthesis commenced with the benzylation of (*R*)-pantolactone **108a** to produce (+)-**109a** in 85% yield. Next, treatment of (+)-**109a** with *N*,*O*-dimethylhydroxylamine hydrochloride, and an excess of MeMgBr followed by immediate TBS ether formation produced (+)-**110a** in 82% yield over 2 steps. Wittig olefination of (+)-**110a** with phosphonate **111** furnished the desired (*E*)-(+)-**112a**, which was then converted into Hatakeyama’s enynol (−)-**82b** using a four-step sequence to complete the formal synthesis of inthomycin C ((+)-**3**). The spectroscopic data as well as the [α]_D_ value of the present compound (−)-**82b** were well-matched with that published previously [22,60]. Similarly, (*S*)-pantolactone **108b** was transformed into enyol (+)-**82b** by following the same procedures as described for (−)-**82b** to accomplish the formal synthesis of inthomycin C ((−)-**3**).

**Scheme 16:**
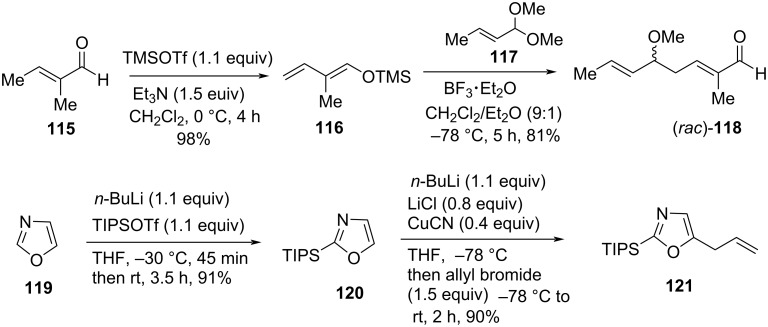
Synthesis of the cross-metathesis precursors (*rac*)-**118** and **121**.

**Scheme 17:**
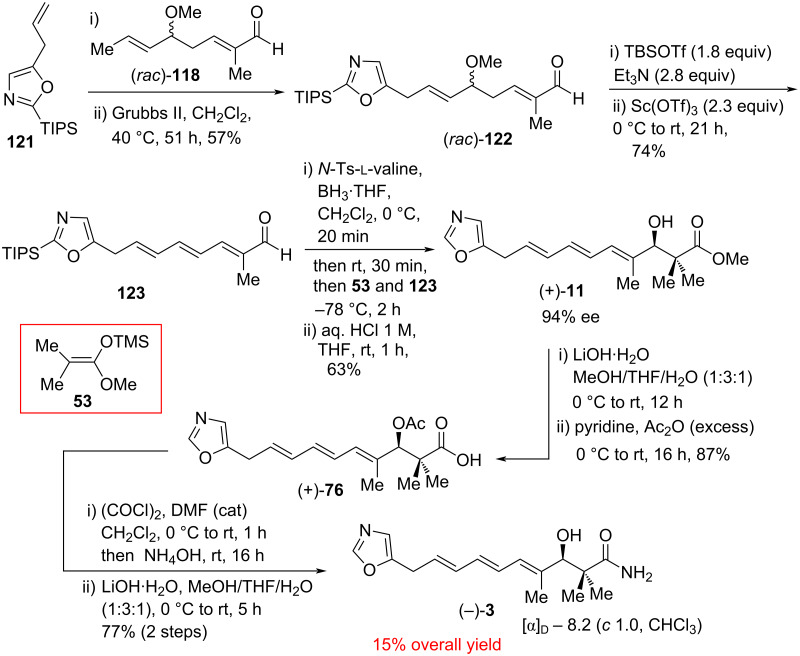
Donohoe’s total synthesis of inthomycin C ((−)-**3**).

In 2018, Donohoe et al. demonstrated a tin-free, short and efficient total synthesis of inthomycin C ((−)-**3**) by comprising the three key steps of C−C bond-forming reactions: i) a vinylogous Mukaiyama aldol reaction, ii) an olefin cross-metathesis reaction, and iii) an asymmetric Mukaiyama–Kiyooka aldol reaction (Scheme 16 and Scheme 17) [8]. The total synthesis was initiated with the preparation of two alkenes precursors (*rac*)-**118** and **121**. The tiglic aldehyde **115** was converted into silyl enol ether **116** followed by treatment with acetal **117** using a vinylogous Mukaiyama aldol reaction to produce the desired aldehyde (*rac*)-**118** in 81% yield. Meanwhile, the TIPS protection of oxazole **119** and then subsequent lithiation at the C-5 position and quenching in situ with allyl bromide furnished oxazole derivative **121** in 90% yield (Scheme 16).

An attempted cross-metathesis reaction of **121** with alkene fragment (*rac*)-**118** in the presence of Grubbs II (G-II) catalyst under optimized conditions produced (*rac*)-**122** in 57% yield. After exhaustive experimentation, demethoxylation of (*rac*)-**122** was achieved to produce (*E,E,E*)-aldehyde **123** predominantly in an 8:1 mixture of diastereoisomers. Then, the key aldol reaction of **123** with silyl enol ether **53** under optimized Mukaiyama–Kiyooka conditions, followed by TIPS deprotection, afforded adduct (3*R*)-(+)-**11** in 63% yield and with 94% ee. Ester hydrolysis followed by acetylation of (3*R*)- (+)-**11** produced acid derivative (+)-**76** [50] in 87% yield. Finally, compound (+)-**76** underwent amidation and deacetylation to give an 11.1:1 mixture of geometrical isomers of inthomycin C ((−)-**3**) in 77% yield over two steps (Scheme 17) [50]. The (3*R*) stereochemistry of (−)-**3** was confirmed by MTPA ester derivatization, which supports the recent work by Hale and Hatakeyama [57].

**Scheme 18:**
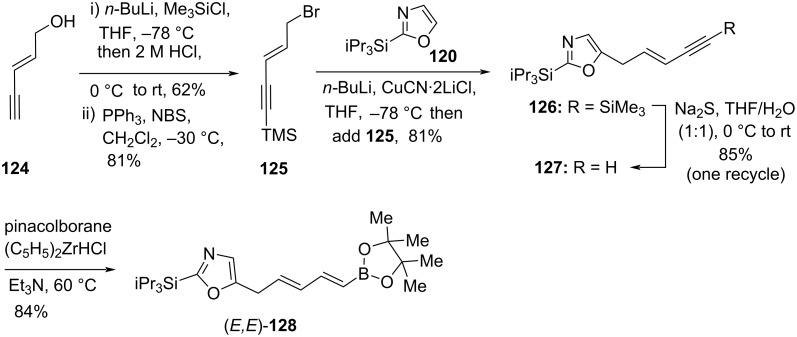
Synthesis of dienylboronic ester (*E,E*)-**128**.

**Scheme 19:**
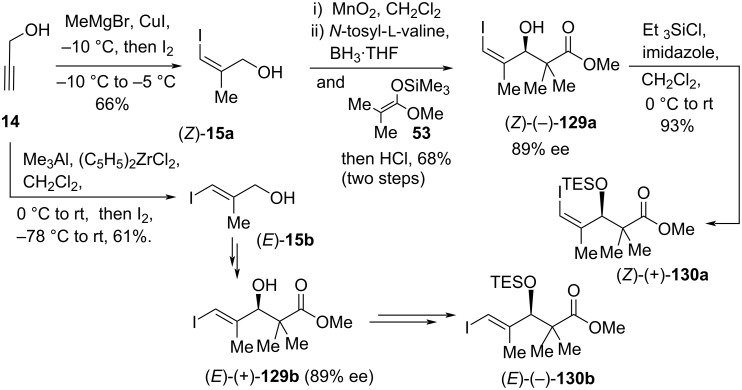
Synthesis of the alkenyl iodides (*Z*)- and (*E*)-**130**.

**Scheme 20:**
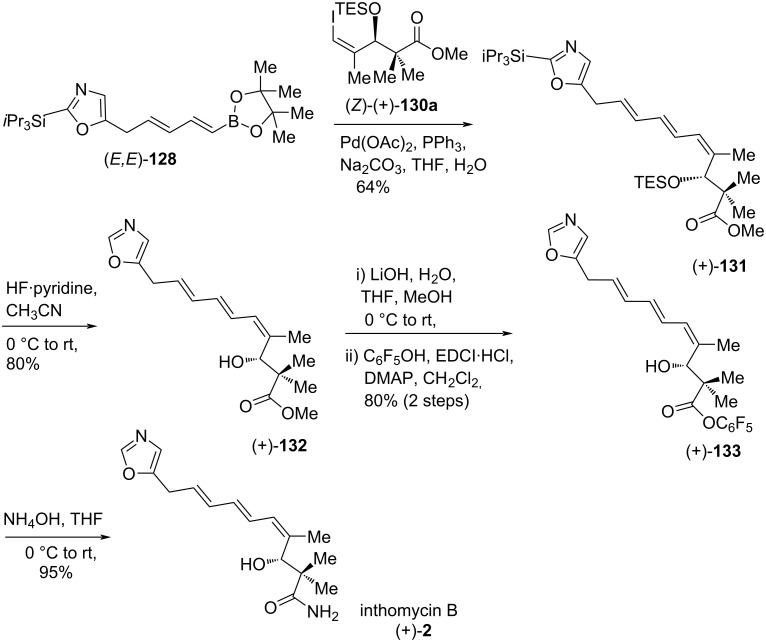
Burton’s total synthesis of inthomycin B ((+)-**2**).

**Scheme 21:**
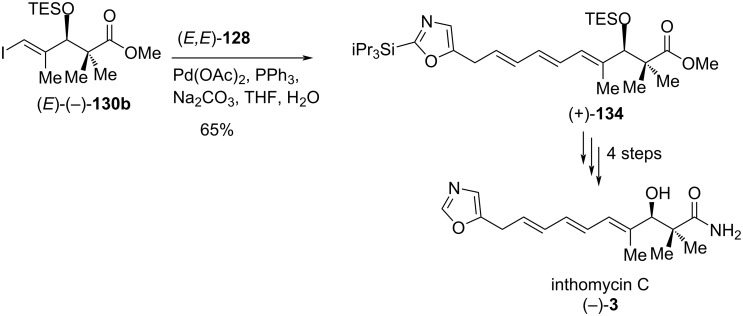
Burton’s total synthesis of inthomycin C ((−)-**3**).

**Scheme 22:**
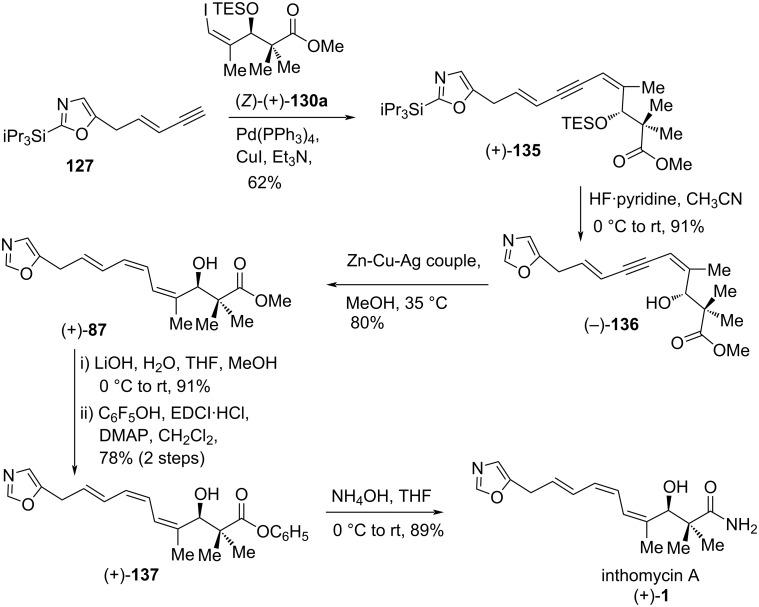
Burton’s total synthesis of inthomycin A ((+)-**1**).

Recently, Burton’s group developed some efficient and tin-free total syntheses of all three inthomycins A–C ((+)-**1**, (+)-**2**, and (−)-**3**) using a Suzuki or Sonogashira cross-coupling of the (*E*)- or (*Z*)-alkenyl iodides **130** with the dienylboronic ester **128** as key step (Schemes 18–22). Initially, (*E*)-pent-2-en-4-yn-1-ol (**124**) was smoothly converted into the desired bromide derivative **125** [67] in two simple steps. The bromide **125** was then reacted with pre-lithiated oxazole derivative **120** [68–69] under optimized conditions to produce coupled product **126**. The selective deprotection of the TMS group of **126** was found to be extremely challenging. The commonly used conditions provided the allene as a major product instead of the desired product. Ultimately, the mono-desilylated product **127** was obtained in 85% yield by using sodium sulfide in a mixture of THF and water. Next, the zirconium-catalyzed hydroboration of the terminal acetylene in **127** gave (*E,E*)-**128** in good yield and with complete stereocontrol (Scheme 18).

To accomplish the key Suzuki coupling of dienylboronic ester **128**, the necessary alkenyl iodides (*Z*)- and (*E*)-**130** were prepared from the propargyl alcohol (**14**) in good yields using a four-step sequence such as Negishi’s (*Z*) and (*E*)-stereoselective isomerization of the terminal alkyne followed by iodinolysis [19,70–71], oxidation to the corresponding aldehydes and enantioselective Kiyooka–Mukaiyama aldol reaction followed by TES protection of the resulting alcohols (Scheme 19).

For the synthesis of inthomycin B ((+)-**2**), a Suzuki coupling reaction between (*E,E*)-**128** and (*Z*)-(+)-**130a** was performed in the presence of palladium(II) acetate, triphenylphosphine, and aqueous sodium carbonate to give (*E,E,Z*)-triene (+)-**131** selectively in 64% yield. Treatment of triene (+)-**131** with HF·pyridine in acetonitrile [22] gave double silyl deprotected triene (+)-**132** which was then converted into inthomycin B ((+)-**2**) via aminolysis of the corresponding pentaflurophenyl ester (+)-**133**. The synthetic inthomycin B ((+)-**2**) showed well-matched spectroscopic properties with that of both natural and synthetic inthomycin B (Scheme 20) [2,22,43].

For the synthesis of inthomycin C ((−)-**3**), the Suzuki coupling between the dienylboronate (*E*,*E*)**-128** and the (*E*)-(−)-**130b** was carried out under optimized conditions to produce the (*E,E,E*)-triene derivative (+)-**134** in 65% yield (Scheme 21). Then, compound (+)-**134** was converted smoothly into inthomycin C ((−)-**3**) by following the similar sequence of reactions as described for the synthesis of inthomycin B ((+)-**2**, see Scheme 20).

Having been successful in the synthesis of inthomycins B ((+)-**2**) and C ((–)-**3**), a significant effort was given to the synthesis of inthomycin A ((+)-**1**). In the beginning, conversion of enyne **127** to the corresponding (*Z,E*)-dienylboronic ester **128** was investigated in the presence of rhodium(I)-catalyzed anti-selective hydroboration [72] under several conditions. Unfortunately, the yield of the desired (*Z,E*)-**128** was found to be poor (<40%). Therefore, enyne **127** was selected as a coupling partner for the key Sonogashira reaction of alkenyl iodide (*Z*)-(+)-**130a** to construct the required (*Z,Z,E*)-triene for completion of the inthomycin A ((+)-**1**) synthesis. Fortunately, the alkenyl iodide (*Z*)-(+)-**130a** coupled smoothly with enyne **127** in the presence of Pd(PPh_3_)_4_, CuI, and triethylamine to give (+)-**135** in 62% yield. The double desilylation of compound (+)-**135** using HF·pyridine in acetonitrile afforded (–)-**136** in 91% yield. The semi-hydrogenation of (–)-**136** to produce the desired (*Z,Z,E*)- triene (+)-**87** was challenging under a variety of conditions, and eventually, it was achieved by the use of the Zn(Cu/Ag) couple in methanol [73]. Finally, the methyl ester (+)-**87** was readily transformed into inthomycin A ((+)-**1)** via pentaflurorophenyl ester (+)-**137** by the same reaction sequence as described for inthomycin B ((+)-**2**) in Scheme 20. In this route, the synthetic inthomycin A ((+)-**1**) was contaminated with a small amount (<10%) of inthomycin B ((+)-**2**, Scheme 22).

**Scheme 23:**
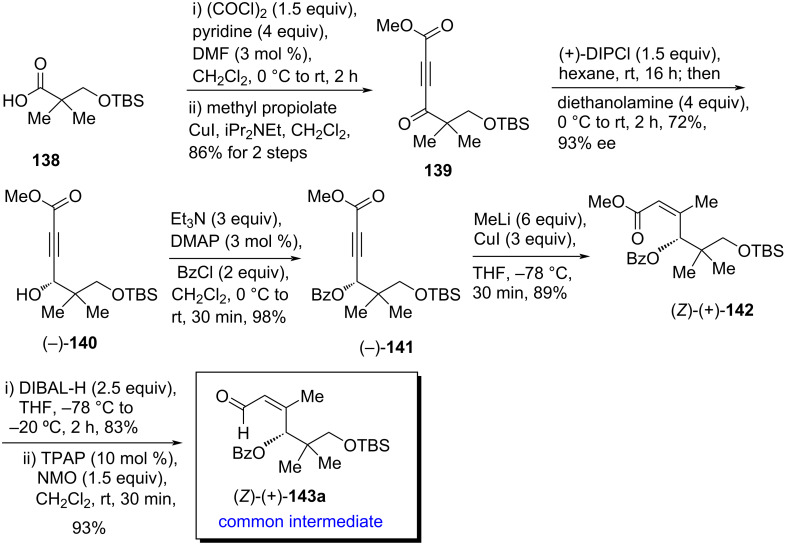
Synthesis of common intermediate (*Z*)-(+)-**143a**.

**Scheme 24:**
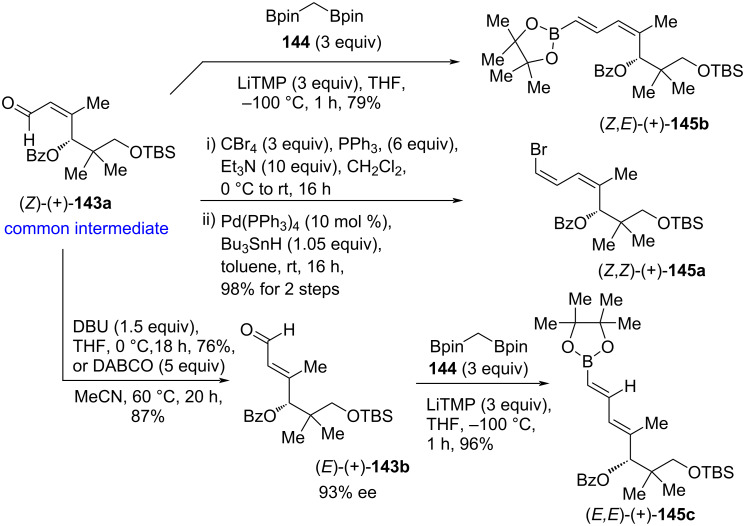
Synthesis of (*Z*)-and (*E*)-selective fragments (+)-**145a–c**.

**Scheme 25:**
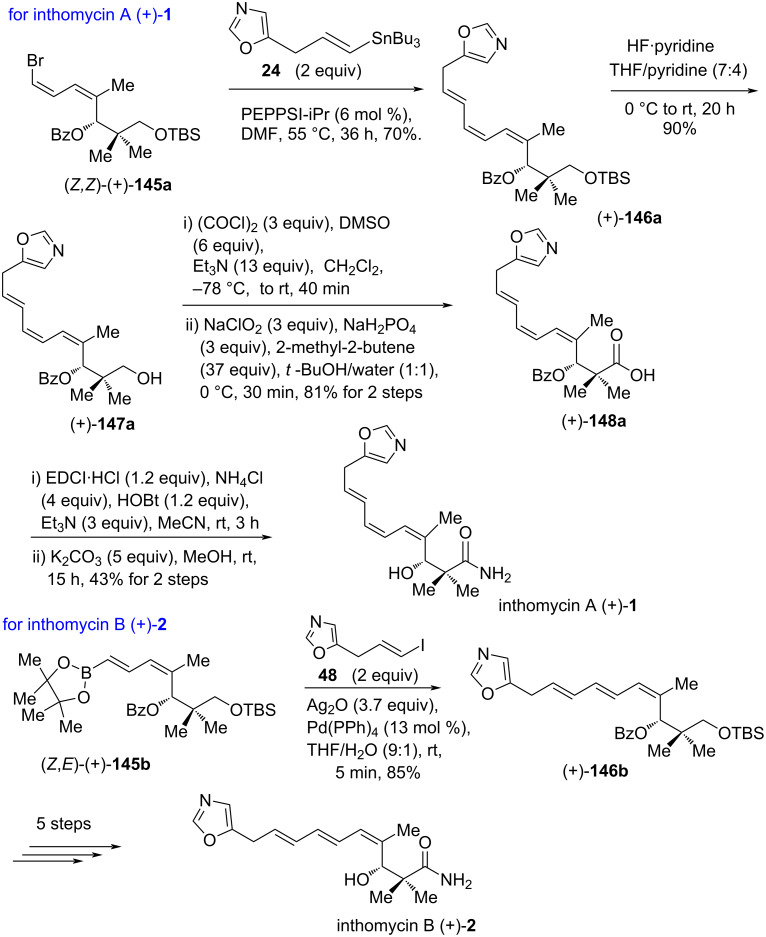
Kim’s total synthesis of inthomycins A (+)-**1** and B (+)-**2**.

**Scheme 26:**
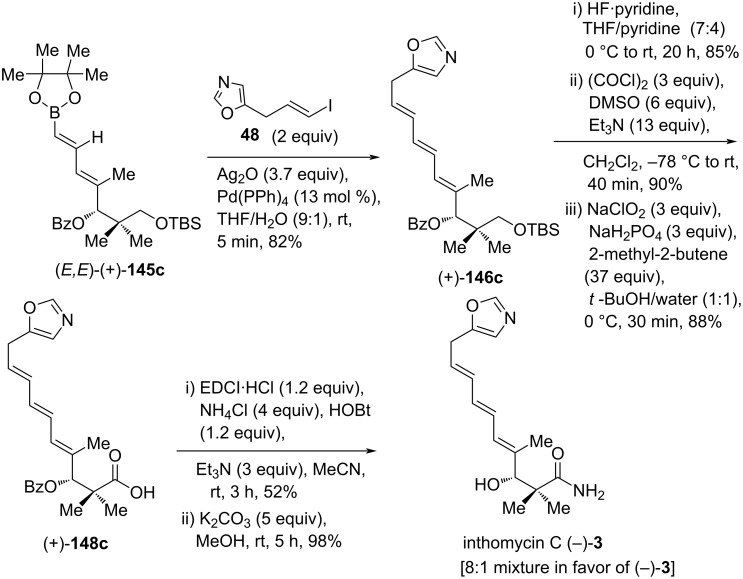
Completion of total synthesis of inthomycin C ((–)-**3**) by Kim.

Very recently, Kim et al. [24] developed a unified strategy to access all the three isomers of inthomycins A–C ((+)-**1**, (+)-**2**, and (−)-**3**) by using a common intermediate (*Z*)-**143a** and an oxazole-derived vinylstannane **24** or vinyl iodide **48** (Schemes 23–26). The synthesis started with the preparation of TBS-protected hydroxy pivalic acid **138** [74], following the literature procedure. The acid **138**, when submitted to the reaction with oxalyl chloride followed by a CuI-catalyzed nucleophilic alkyne addition of methyl propiolate, provided ynone **139** in 86% yield over two steps. When, compound **139** was treated with (+)-diisopinocampheylchloroborane (DIPCl) [75–76] at room temperature and the resulting mixture was processed as in the usual manner using diethanolamine, the expected alcohol (−)-**140** was obtained in 93% ee and 72% yield. Compound (−)-**140** was further transformed to the corresponding Mosher's esters and their NMR and X-ray crystallographic data were well-matched with the (*R*)-stereostructure of (−)-**140** [77–78]. The hydroxy group of (−)-**140** was protected as benzoate to give (−)-**141** in good yield. Next, the benzoate-protected ynoate (−)-**141** was converted into aldehyde (*Z*)-(+)-**143a** by employing a copper-catalyzed methylation of the alkyne moiety to the corresponding enoate (*Z*)-(+)-**142** followed by an ester reduction–oxidation sequence using DIBAL-H and TPAP (Scheme 23).

The aldehyde (*Z*)-(+)-**143a** was the common intermediate for the synthesis of (*E*)- or (*Z*)-selective vinyl boronates or vinyl halides (+)-**145** to accomplish total syntheses of all inthomycins A–C ((+)-**1**, (+)-**2** and (–)-**3**, see Scheme 25 and Scheme 26). With the key intermediate (*Z*)-(+)-**143a** in hand, it was transformed into (*E*)-vinylboronate (+)-**145b** in perfect stereoselectivity by using the recently developed boron-Wittig reaction with bis[(pinacolato)boryl]methane (**144**) [79]. By applying the Corey–Fuchs dibromoolefination and followed by Pd-catalyzed hydrogenolysis under Uenishi's conditions [80], compound (*Z*)-(+)-**143a** delivered the bromodiene (*Z,Z*)-(+)-**145a** in good yield and stereoselectivity. Isomerization of (*Z*)-(+)-**143a** to (*E*)-(+)-**143b** was carried out successfully using sterically hindered base DBU or DABCO. Treatment of (*E*)-(+)-**143b** with **144** using the boron-Wittig reaction [79] afforded the desired (*E,E*)-vinylboronate (*E*,*E*)-(+)-**145c** in 96% yield (Scheme 24).

Followed by the successful access to (*Z*)- and (*E*)-selective isomers of (+)-**145a–c**, further exploration to the incorporation of a triene unit of inthomycins was investigated. The PEPPSI-iPr-catalyzed [58] Stille cross-coupling between vinyl bromide (*Z,Z*)-(+)-**145a** and (*E*)-vinylstannane **24** [43] proceeded without significant isomerization to give (4*Z*,6*Z*,8*E*)-triene (+)-**146a**, a subunit of inthomycin A ((+)-**1**). Meanwhile, the Suzuki–Miyaura coupling of vinylboronate (*Z,E*)-(+)-**145b** with the known (*E*)-oxazole iodide **48** [43] was performed successfully to afford geometrically pure (4*Z*,6*E*,8*E*)-triene (+)-**146b** in 85% yield following the synthesis of inthomycin B ((+)-**2**). Careful deprotection of silyl ether of (+)-**146a** with HF∙pyridine in THF/pyridine delivered alcohol (+)-**147a**. Next, the Swern oxidation of the resulting alcohol (+)-**147a** followed by Pinnick oxidation afforded the acid (+)-**148a**. Finally, the EDCl-mediated amidation of unstable acid (+)**-148a** followed by debenzoylation produced inthomycin A ((+)-**1**). Similarly, compound (+)-**146b** was converted into inthomycin B ((+)-**2**) (Scheme 25).

To complete the total synthesis of inthomycin C ((−)-**3**), a cross-coupling between (*E,E*)-(+)-**145c** and vinyl iodide **48** was carried out to produce (4*E*,6*E*,8*E*)-triene (+)-**146c** in 82% yield. Utilizing the same sequence as applied to the synthesis of inthomycin As ((+)-**1**) and B ((+)-**2**) from (+)-**146a** and (+)-**146b**, respectively (see Scheme 25), the triene (+)-**146c** was successfully converted into an 8:1 mixture of inthomycin C ((−)-**3**) and another minor isomer in good overall yield after the final step (Scheme 26). The spectroscopic data and specific rotation values of the three inthomycins A–C ((+)-**1**, (+)-**2**, and (−)-**3**) were consistent with those reported previously [8,23,57]. The absolute configurations of inthomycins A–C ((+)-**1**, (+)-**2**, and (−)-**3**) were reconfirmed as 3*R* by assigning the (*R*)-stereochemical descriptor for the common intermediate (−)-**140**, which supports the recent work of Hale and Hatakeyama [57].

## Conclusion

This review highlighted reports on the various synthetic efforts for both the formal and total synthesis of racemic and enantiopure inthomycins A–C (**1**–**3**). These compounds have three key structural features: an oxazole ring, a triene system, and an amide moiety with a chiral, hydroxylated carbon at the β-position. These interesting structures accompanied by their promising biological activities and the lack of natural sources have made inthomycins an attractive target in the synthetic organic community to work intensively in this area. The synthesis of simple looking inthomycins is challenging due to the unusually interposed functional groups and isomerizable double bonds in the conjugated triene moiety. Various stereoselective cross-coupling reactions such as Stille, Suzuki, or Sonogashira or Suzuki–Miyaura have been utilized to construct the geometrically distinctive polyene systems of inthomycins A–C (**1**–**3**). The elegant work of R. J. K. Taylor [21,43], Ryu [50], Donohoe [57], and Burton [23] demonstrated the power of the Mukaiyama−Kiyooka aldol reactions to install the asymmetric center of inthomycins. Alternatively, Hatakeyama and Kim’s groups employed an asymmetric β-lactone synthesis and an asymmetric ynone reduction protocol for the construction of the stereogenic center of inthomycins, respectively [22,24]. Despite these recent advances, the development of novel methods for the regio- and stereocontrolled synthesis of inthomycins, inthomycin-embedded natural products, and their synthetic analogues with better biological outcomes is of strategic importance and being continued for further discovery.
